# Mosquito prevalence, resting habitat preference, and *Plasmodium* infection status of anophelines in coastal Karnataka during the declining phase of malaria—an exploratory study

**DOI:** 10.1007/s00436-024-08322-x

**Published:** 2024-08-22

**Authors:** Gowthami Arumugam, Kavitha Saravu, Prashanth Kotthapalli, Vishnu Teja Nallapati, Prashanth Bhat, Muktha Achari, Naveenchandra Kulal, Shriram Ananganallur Nagarajan, Hoti S. L., Ashwani Kumar

**Affiliations:** 1 Department of Infectious Diseases, Kasturba Medical College, Manipal, Manipal Academy of Higher Education, Manipal, Karnataka India 576104; 2 Department of Health and Family Welfare, Government of Karnataka, Udupi District, India; 3 Department of Health and Family Welfare, Government of Karnataka, Dakshina Kannada District, India; 4 ICMR-Vector Control Research Centre, Puducherry, India; 5 Centre for Global Health Research, Saveetha Medical College and Hospital, Saveetha Institute of Medical and Technical Sciences, Saveetha University, Chennai, 602105 Tamil Nadu India

**Keywords:** *Anopheles*, Malaria, PCR, Resting preferences, India

## Abstract

Malaria has a historical presence in the Dakshina Kannada (D.K.) and Udupi districts of Karnataka, India. To understand the potential involvement of anopheline fauna in malaria transmission, we conducted an exploratory entomological survey. The study is crucial given the decreasing malaria incidence in these districts in recent years. From September 2022 to August 2023, we collected indoor resting mosquitoes using a manual aspirator at 27 randomly chosen sites within three distinct resting habitats (human dwellings, cattle sheds, and construction sites) in the urban areas of Udupi and Dakshina Kannada districts. Mosquitoes were morphologically identified, and anopheline specimens were tested for the presence of malarial parasite by polymerase chain reaction (PCR) analysis. We collected a total of 1810 mosquitoes, comprising 21 species distributed across five genera. *Culex* emerged as the predominant genus, constituting 84.4% of the collected specimens, while *Anopheles* accounted for 5.4%. Among the observed species, *Culex quinquefasciatus* was predominant, comprising 77.9% of the mosquito specimens collected in this study. Two malaria vectors, *An. stephensi* and *An. subpictus* complex, constituted 16.3% and 1.0% of the total anophelines collected, respectively. None of the 96 female anophelines was tested positive for *Plasmodium* infection. Our findings suggest that *Anopheles* mosquitoes prefer resting in cattle sheds over human dwellings. While our study identified two malaria vectors, they were present at low densities. To gain a more comprehensive understanding of the dynamics of these vector mosquitoes, it is essential to conduct long-term surveillance to monitor their prevalence and role in malaria transmission.

**Supplementary Information:**

The online version contains supplementary material available at 10.1007/s00436-024-08322-x.

## Introduction

Malaria, a parasitic disease transmitted by *Plasmodium*-infected female *Anopheline* mosquitoes, poses a significant global health challenge. In 2022, the world witnessed an alarming 249 million reported cases of malaria, resulting in 608,000 deaths (World Health Organization 2023). Within the Southeast Asia region, 5.2 million cases were documented, with India alone contributing 65.7% of these cases in the same year (World Health Organization 2023). Despite India’s substantial malaria burden, there has been a noteworthy decline in malaria cases over the past 15 years (NCVBDC 2023). Specifically, in Karnataka, a south Indian state, a remarkable reduction in malaria morbidity from 109,118 in 2001 to a mere 974 cases in 2021 was seen (Chalageri et al. 2023; Krishna and Haradanhalli 2017). Within Karnataka, Dakshina Kannada (D.K.) and Udupi districts contribute significantly to the major burden of malaria, with the cases primarily located in urban areas (Chalageri et al. 2023; Dayanand et al. 2017; Malaria Elimination Plan in Karnataka 2016). Historically, the rise in malaria cases and mosquito populations in this region has been closely linked to tropical climate, road development projects, uncontrolled urban expansion, multi-storeyed housing complex, population growth, and environmental/ecological changes (Dayanand et al. 2017).

Out of the 537 known species of *Anopheles* mosquitoes worldwide (Harbach 2013), only a subset of approximately 70 to 80 species possesses the capability to transmit malaria to humans (Robert et al. 2011). In the Indian context, there are a total of 58 anopheline species, with 10 of them having a role in malaria transmission within different geographical areas (Baghel et al. 2009; Dev and Sharma 2013; Subbarao et al. 2019). Notably, *Anopheles stephensi*, an urban dweller, stands out as a major malaria vector, even though its presence is often observed at relatively low densities (Subbarao et al. 2019). This mosquito species is significantly responsible for the transmission of urban malaria, particularly in the state of Karnataka (Anvikar et al. 2016; Ghosh et al. 2008). It breeds in stagnant freshwater, including overhead tanks, cemented storage tanks, cisterns, peri-domestic containers, curing waters, and sunken pits commonly found at construction sites (Anvikar et al. 2016; Dash et al. 2007).

The government of Karnataka has implemented vector control measures aimed at interrupting the transmission of urban malaria in the selected endemic zones. These measures involve the distribution of long-lasting insecticide-treated nets (LLINs), the use of larvicides, and the introduction of guppy and *Gambusia* fish in the community (Chalageri et al. 2023; Malaria Elimination Plan in Karnataka (2016 to 2025). As malaria transmission intensity has decreased owing to these efforts (Chalageri et al. 2023), there arises a greater need to monitor the transmission potential and relative abundance of anopheline mosquitoes.

Furthermore, there may exist other neglected mosquito species that could contribute to malaria transmission at lower levels within the study area. Consequently, gathering such information through entomological surveillance becomes essential to envisage effective vector control strategies aimed at eliminating malaria. It is pertinent to state that research on mosquito diversity in Karnataka has been relatively scanty (Ganesh et al. 2004; Ishwara Prasad et al. 2021; Kanojia 2007; Kanojia and Jamgaonkar 2008; Prasad and Sreepada 2013; Rajavel et al. 2006; Shetty et al. 2007; Urmila et al. 1999), and our understanding of mosquito populations in the urban and coastal landscapes of this region remains inadequate. Hence, we conducted a pilot study to shed light on the composition of anopheline mosquito population and their potential role in disease transmission in two coastal areas that have historical association with malaria.

## Material and methods

### Study area

The coastal districts of Udupi and Dakshina Kannada (D.K.) are located approximately 30 km from the western shore of the Arabian Sea, separated from the interior by the lush western ghats to the east. Mangalore (12° 54ʹ 56.1780ʺ N, 74° 51ʹ 21.474ʺ E), the administrative centre of D.K. district, is a prominent coastal city characterised by a densely populated metropolitan area of 170 km^2^, primarily comprising residential and commercial buildings. Udupi town occupies 68.23 km^2^ (13° 20ʹ 27.1716ʺ N, 74° 44ʹ 31.7112ʺ E), situated approximately 58 km north of Mangalore in Udupi district, and holds a special place as a pilgrimage centre.

Both coastal cities are renowned for their rich and ancient culture, marked by numerous festivals, traditional celebrations, and year-round concerts. However, they are also considered as high endemic areas for malaria transmission due to rapid economic expansion and urbanization. Further, these two districts experience heavy rainfall between June and September due to southwest monsoon and exhibit warm-humid tropical climate conditions conducive to mosquito proliferation on an annual basis. The average annual rainfall in the study area was 3125 mm, with an average humidity of 83% during the study period.

### Resting mosquito collection

An entomological survey focusing on anopheline fauna distribution was conducted at two densely populated urban cities, Udupi and Mangalore, in monsoon (May–Oct) and non-monsoon (Nov–April) seasons during the year 2022 to 2033. The study involved sampling of adult resting mosquitoes from three distinct habitats—human dwellings, construction sites, and cattle sheds at 27 sampling points within the study area. A construction site is defined as a place where building structures are actively being constructed, which creates temporary water bodies and provides dark shady areas that serve as ideal conditions for mosquito proliferation. Of the 27 sampling points, 13 were randomly selected from Udupi and 14 from Mangalore, in which 10 human dwellings and 2 construction sites were chosen at each sampling points. However, the same resting habitats were selected for both seasons, and the cattle sheds were surveyed randomly based on their availability and accessibility on the study area.

The survey involved random or on regular inspections of resting habitats across the two seasons, with each session lasting 15 to 30 min. The mosquito collections were conducted at the selected sampling points once per season during the dawn hours (from 5.30 a.m. to 8 a.m.) and evening hours (6 p.m. to 8.30 p.m.), using a manual aspirator and a flashlight. Additionally, location data, including geographical coordinates and altitude, were recorded using the geo Tracker mobile application (Version: 5.3.4.3910), and a map was generated using Q-GIS software (Version: 3.36.3).

### Laboratory processing

The mosquito specimens collected from the field were etherised and then subjected to species identification through direct observation of the morphological characters using a stereomicroscope, following standard taxonomic keys (Nagpal et al. 2005; World Health Organization 2020).

The abdominal conditions of female mosquitoes were recorded separately, classifying them as unfed (UF), freshly fed (FF), semi-gravid (SG), or gravid (G). Subsequently, anopheline specimens were categorised by species, depending on the collection sites. They were placed in 1.5-ml Eppendorf tubes and dried for 12 h at 90 °C. The dried specimens were then sealed in Ziplock bag with silica gel and transported to the ICMR-VCRC laboratory, Pondicherry.

The body parts of individual mosquitoes were separated into head + thorax and abdomen for DNA extraction. A maximum of five mosquito body parts of head + thorax or abdomen were pooled, and placed in separate 1.5-ml Eppendorf tubes, yielding 20 pools. DNA was extracted according to the manufacturer’s protocol based on QIAamp DNA Mini Kit (QIAGEN GmbH, Hilden, Germany). Following extraction, DNA amplification was performed to detect *Plasmodium* infection, using primers (S1 File) and methodology described by Snounou et al. (1993). Gel electrophoresis and image visualization process were done as previously described elsewhere (Kumar et al. 2012; Sahu et al. 2017). Additionally, the per–man hour density (PMHD) was calculated for each *Anopheles* mosquito species, representing the number of female mosquitoes collected per man hour spent on collection.

### Data analysis

The collected data were entered into Microsoft Excel and analysed using GraphPad Prism version 5 for Windows. In term of descriptive analysis, the number of mosquitoes in the study areas was expressed using percentages and mean ± standard error (SE). The data underwent Shapiro–Wilk normality test, and for non-normal distributed data, the Mann–Whitney *U* test was employed to assess the variations in mosquito abundance between the two seasons. Kruskal–Wallis test was applied to compare the mean densities of *Anopheles* mosquitoes across different resting habitats. Dunn’s pair-wise comparison test was utilised to analyse differences within the habitats. To determine if there was a significant relationship between districts and the prevalence of mosquito densities across genera, Chi-square (*χ*^2^) test was employed. All statistical tests were conducted at the *p* < 0.05 significant level.

## Results

### Species composition

**Fig. 1:**
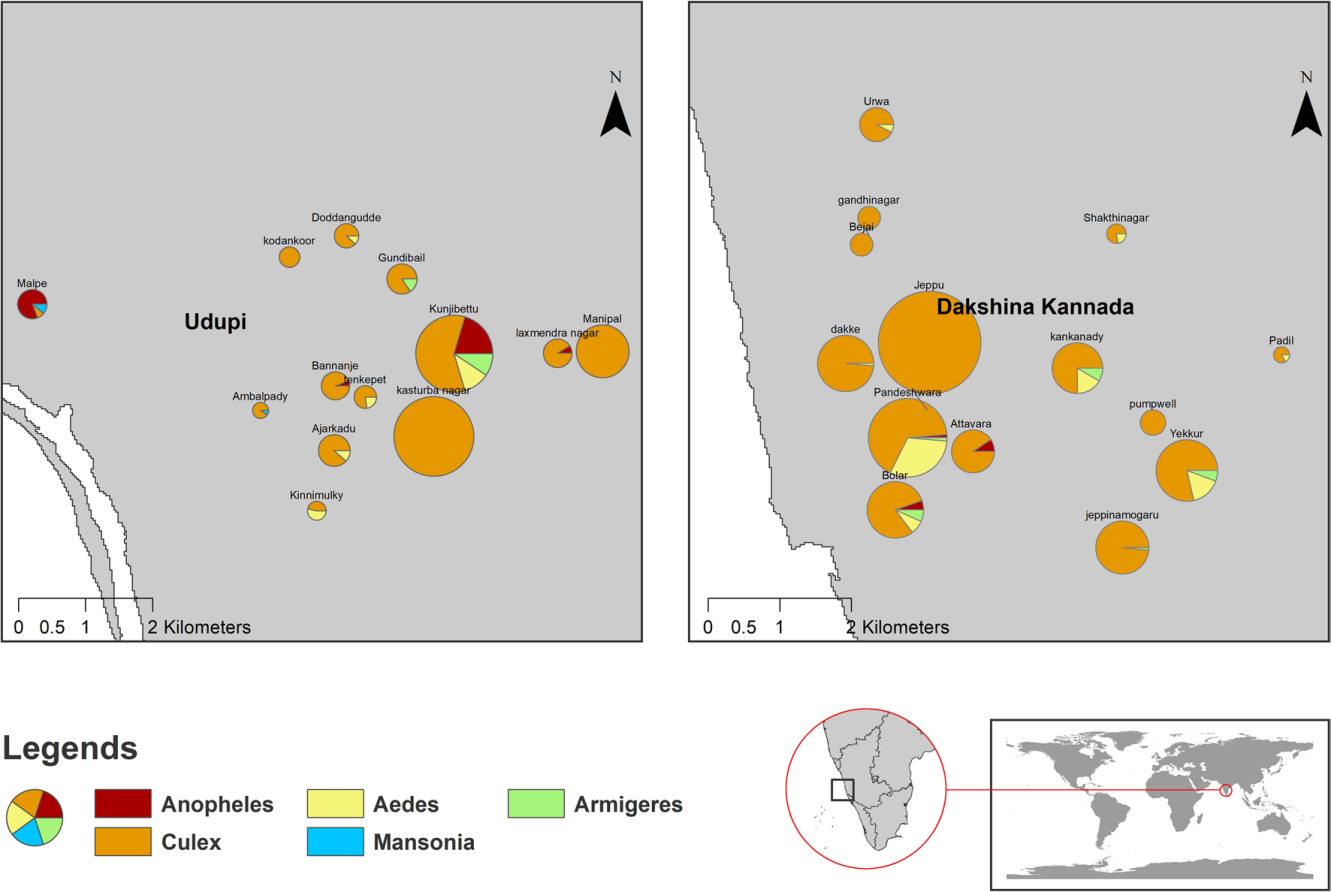
Site wise mosquito collection in two districts of Karnataka (Sep 2022–Aug 2023)

During the study period, spanning from September 2022 to August 2023 and encompassing two seasons, a total of 1810 adult mosquitoes were sampled across 27 different sampling points within two coastal cities in Karnataka, India, situated in the Western Ghats region. Theses mosquitoes were classified into 21 species belonging to five genera. Notably the areas, Kasturba Nagar, Kunjibettu, and Jeppu recorded a higher number of mosquitoes compared to other locations, constituting 15.1%, 14%, and 13.6% of the total, respectively. Additionally, the anopheline mosquitoes were identified in seven of the sampled areas, adding to the overall diversity observed during the study (Fig. 1).

Among five genera, *Culex* emerged as the most predominant genus, comprising a significant 84.4% of the total specimens, with seven species belonging to subgenus *Culex*. In contrast, *Anopheles* accounted for 5.4%, encompassing nine species distributed across the subgenera *Anopheles* and *Cellia*. The subgenus *Stegomyia* represented 7.2% with three species, while *Mansonia* and *Armigeres* were sparsely represented by a single species each in the current study (Table 1).

**Table 1:** Diversity of mosquito fauna of Udupi and Mangalore from Sep 2022 to Aug 2023

Genera	Mosquito species	Udupi	Mangalore	S1^a^	S2^b^	N^c^	%^d^	PMHD^e^
*Anopheles*	*Anopheles (Anopheles) nigerrimus* Giles 1900	√	˟	3	10	13	0.72	5.2
	*Anopheles (Anopheles) barbirostris* s.l. Van der Wulp 1984	√	√	5	12	17	0.94	6.8
	*Anopheles (Anopheles) peditaeniatus* Leicester 1908	√	˟	1	2	3	0.17	1.2
	*Anopheles (Cellia) jamesii* Theobald 1901	√	˟	3	20	23	1.27	9.2
	*Anopheles (Cellia) pseudojamesii* Strickland 1927	√	˟	0	5	5	0.28	2
	*Anopheles (Cellia) subpictus* Grassi 1899	√	˟	0	1	1	0.06	0.4
	*Anopheles (Cellia) vagus* Doenitz 1902	√	˟	9	6	15	0.83	6
	*Anopheles (Cellia) tessellatus* Theobald 1901	√	˟	0	5	5	0.28	2
	*Anopheles (Cellia) stephensi* Liston 1901	√	√	13	3	16	0.88	6.4
*Culex*	*Culex (Culex) quinquefasciatus* Say 1823	√	√	462	948	1410	77.90	5640
	*Culex (Culex) vishnui* Theobald 1901	√	√	38	9	47	2.60	18.8
	*Culex (Culex) pesudovishnui* Theobald 1902	√	˟	6	0	6	0.33	2.4
	*Culex (Culex) tritaeniorhynchus* Giles 1901	√	˟	7	0	7	0.39	2.8
	*Culex (Culex) gelidus* Theobald 1901	√	√	53	0	53	2.93	21.2
	*Culex (Culex) whitmori* Giles 1904	√	˟	0	2	2	0.11	0.8
	*Culex (Culex) fuscocephala* Theobald 1907	√	˟	3	0	3	0.17	1.2
*Aedes*	*Aedes (stegomyia) albopictus* Kuse 1894	√	√	42	20	62	3.43	24.8
	*Aedes (stegomyia) aegypti* Linnaeus 1762	˟	√	45	3	48	2.65	19.2
	*Aedes (stegomyia) vittatus* Bigot 1861	√	˟	20	0	20	1.10	8
*Mansonia*	*Mansonia (Mansonioides) uniformis* Theobald 1901	√	˟	0	5	5	0.28	2
*Armigeres*	*Armigeres (Armigeres) subalbatus* Coquillett 1898	√	√	36	13	49	2.71	19.6

√Mosquitoes captured in the study area; ˟Mosquitoes not captured in the study area; ^a^number of mosquitoes collected in monsoon season; ^b^number of mosquitoes collected in non-monsoon season; ^c^total no. of mosquitoes collected; ^d^percentage of mosquitoes collected; ^e^per man hour density

Of the 1810 mosquitoes collected, *Culex quinquefasciatus* was the most prevalent species, constituting a 77.9% of all specimens, followed by *Aedes albopictus* (3.43%). In contrast, *Culex whitmori* and *Anopheles subpictus* complex were the least collected species, accounting for 0.11% and 0.06% respectively in the present study. Notably, the species common to both areas included *An. barbirostris* s.l., *An. stephensi*, *Cx. quinquefasciatus*, *Cx. vishnui*, *Cx. gelidus*, *Ae. albopictus*, and *Armigeres subalbatus.* The mosquito density of *Anopheles*, *Culex*, and *Mansonia* genera were higher during the non-monsoon period (*n* = 1028), in comparison to the monsoon season (*n* = 603). In contrast, genera *Aedes* and *Armigeres* density were higher during the monsoon period (*n* = 143), compared to the non-monsoon period (*n* = 36). The diversity of mosquito fauna of Udupi and Mangalore is summarized in Table 1.

**Fig. 2:**
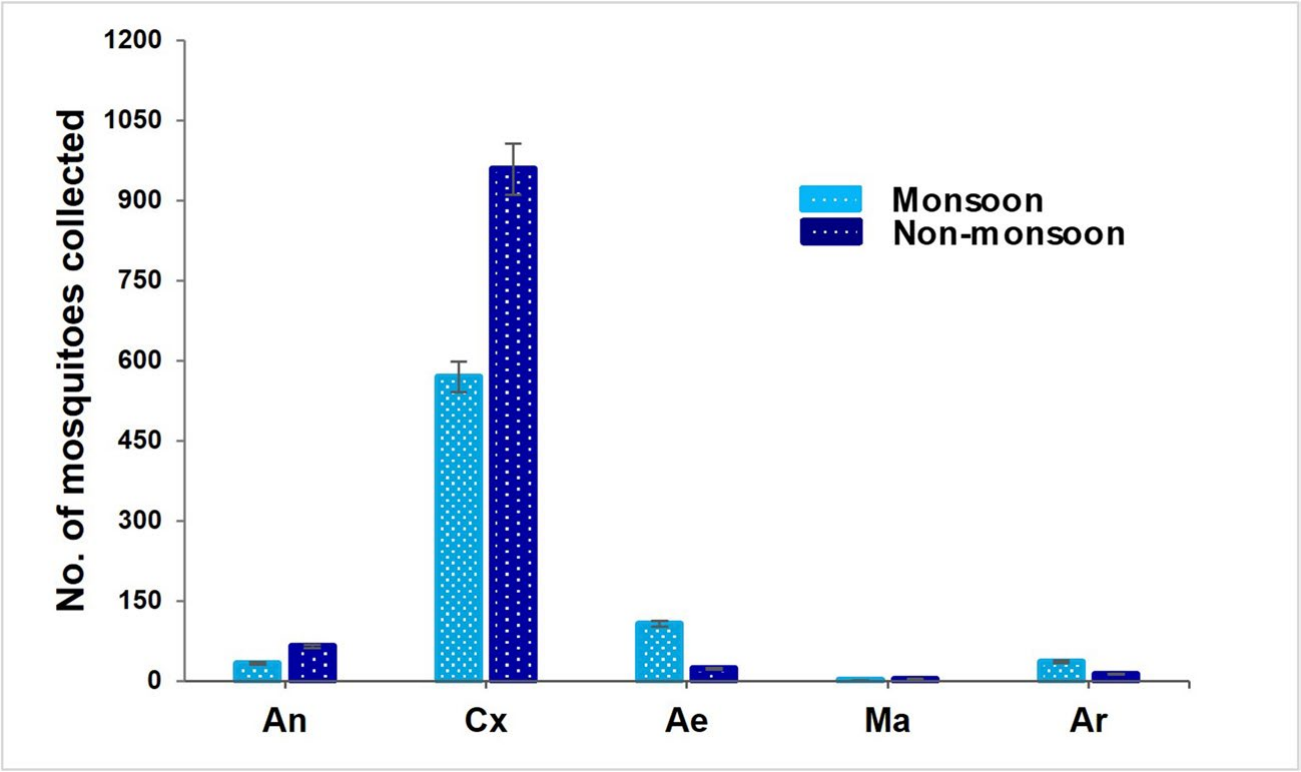
Number of mosquitoes collected genus-wise during monsoon and non-monsoon seasons in Coastal Karnataka, India (Sep 2022–Aug 2023)

The *χ*^2^ test results indicated a significant variation in mosquito density between the two seasons (*P* < 0.0001) among different genera. The graphical representation of mosquitoes collected across two seasons among different genera is illustrated in Fig. 2.

### Anopheline composition and seasonal abundance

A total of 98 *Anopheles* mosquitoes representing nine species were collected and identified. Among the collected species, *An. jamesii*, *An. barbirostris* s.l., *An. stephensi* were the most abundant species, with PMHD of 9.2, 6.8, and 6.4, respectively. In contrast, *An. subpictus* complex was the least collected species with a PMHD of 0.4. Moreover, the number of *Anopheles* mosquitoes collected during the non-monsoon season (66.3%, 95% CI: 0.51 to 0.85) was notably higher than in the monsoon season (33.7%, 95% CI: 0.23 to 0.47) (Table 1) (Fig. 2), and there was a significant difference in mosquito density between the seasons in the study area (*P* = 0.036).

The mosquito density peaked in December 2022 and February 2023, while the remaining months exhibited lower abundance. However, no anophelines was recorded in March and April 2023. Comparably, in terms of *Anopheles* species, the numbers varied across both seasons. For instance, *An. pseudojamesii* (*n* = 5), *An. subpictus* (*n* = 1), and *An. tessellatus* (*n* = 5) were observed only during the non-monsoon season, whereas the remaining six species were observed in both seasons (Table 2).

**Table 2:** Monthly anopheline density variation recorded from Sep 2022 to Aug 2023

	Anopheline mosquitoes								
Months	*An. jamesii* *N* (%)	*An. pseudojamesii* *N* (%)	*An. nigerrimus* *N* (%)	*An. barbirostris* s.l *N* (%)	*An. peditaeniatus* *N* (%)	*An. subpictus* *N* (%)	*An. vagus* *N* (%)	*An. tessellatus* *N* (%)	*An. stephensi* *N* (%)
Sep 22	1 (4.35)	-	1 (7.7)	3 (17.6)	-	-	-	-	2 (12.5)
Oct 22	2 (8.7)	-	2 (15.4)	2 (11.8)	1 (33.3)	-	-	-	-
Nov 22	-	-	-	-	-	1 (100)	-	-	-
Dec 22	14 (60.9)	5 (100)	4 (30.8)	5 (29.4)	2 (66.7)	-	5 (33.3)	5 (100)	-
Jan 23	-	-	-	-	-	-	-	-	4 (25)
Feb 23	6 (26.1)	-	6 (46.2)	7 (41.2)	-	-	1 (6.7)	-	-
Mar 23	-	-	-	-	-	-	-	-	-
Apr 23	-	-	-	-	-	-	-	-	-
May 23	-	-	-	-	-	-	-	-	2 (12.5)
Jun 23	-	-	-	-	-	-	9 (60)	-	3 (18.75)
Jul 23	-	-	-	-	-	-	-	-	4 (25)
Aug 23	-	-	-	-	-	-	-	-	1 (6.25)
Total	23	5	13	17	3	1	15	5	16

*N* Number of mosquitoes collected; *%* percentage of mosquitoes

The mean density of all anophelines collected in Udupi was notably high (3.4 ± 1.4) compared to Mangalore (0.4 ± 0.2) across both monsoon and non-monsoon seasons. However, the average density of the known malaria vector *An. stephensi*, remained around 0.3 ± 0.1 per district, and there was no significant difference between the two districts (*P* = 0.79). Similarly, there was no significant difference in overall anopheline density between the two districts (*P* = 0.76) (Table 3).

**Table 3:** Comparative analysis of anopheline mosquito density among resting habitats in two districts

Mosquito species	Mean ± SE		
	Udupi	Mangalore	Total
Anopheline spp.			
H.D^a^	0.4 ± 0.2	0	0.2 ± 0.1
Con.S^b^	0.3 ± 0.3	0.5 ± 0.4	0.4 ± 0.2
C.SD^c^	13.5 ± 4.0	2.0 ± 2.0	10.6 ± 3.5
All 3 habitats	3.4 ± 1.4	0.4 ± 0.2	2.0 ± 0.8
*p* value	0.0002*	0.11	0.0001*
*Anopheles stephensi*			
H.D^a^	0.3 ± 0.2	0	0.2 ± 0.1
Con.S^b^	0.3 ± 0.3	0.5 ± 0.4	0.4 ± 0.2
C.SD^c^	0.3 ± 0.3	3.0 ± 3.0	0.3 ± 0.2
All 3 habitats	0.3 ± 0.1	0.3 ± 0.2	0.3 ± 0.1
*p* value	0.78	0.21	0.79

^a^Human dwelling

^b^Construction site

^c^Cattle shed

^*^Significant (*p* < 0.05) for Kruskal–Wallis test

Concerning resting habitats, a significant proportion of anophelines were found in the cattle sheds of Udupi (13.5 ± 4.0) compared to human dwellings (0.4 ± 0.2) and construction sites (0.3 ± 0.3). In Mangalore, the proportion of anophelines from construction sites and cattle sheds were 0.5 ± 0.4 and 2.0 ± 2.0 respectively, whereas none were observed in human dwellings. Additionally, the mean density of *An. stephensi* was slightly higher at construction sites (0.4 ± 0.2) than in cattle sheds (0.3 ± 0.2) and human dwellings (0.2 ± 0.1). The Kruskal–Wallis test revealed that the overall *Anopheles* density differed significantly among the different resting habitats (*P* < 0.0001) (Table 3 and S2 File). However, comparison test revealed no significant differences between human dwellings and construction sites (*P* < 0.79).

**Fig. 3:**
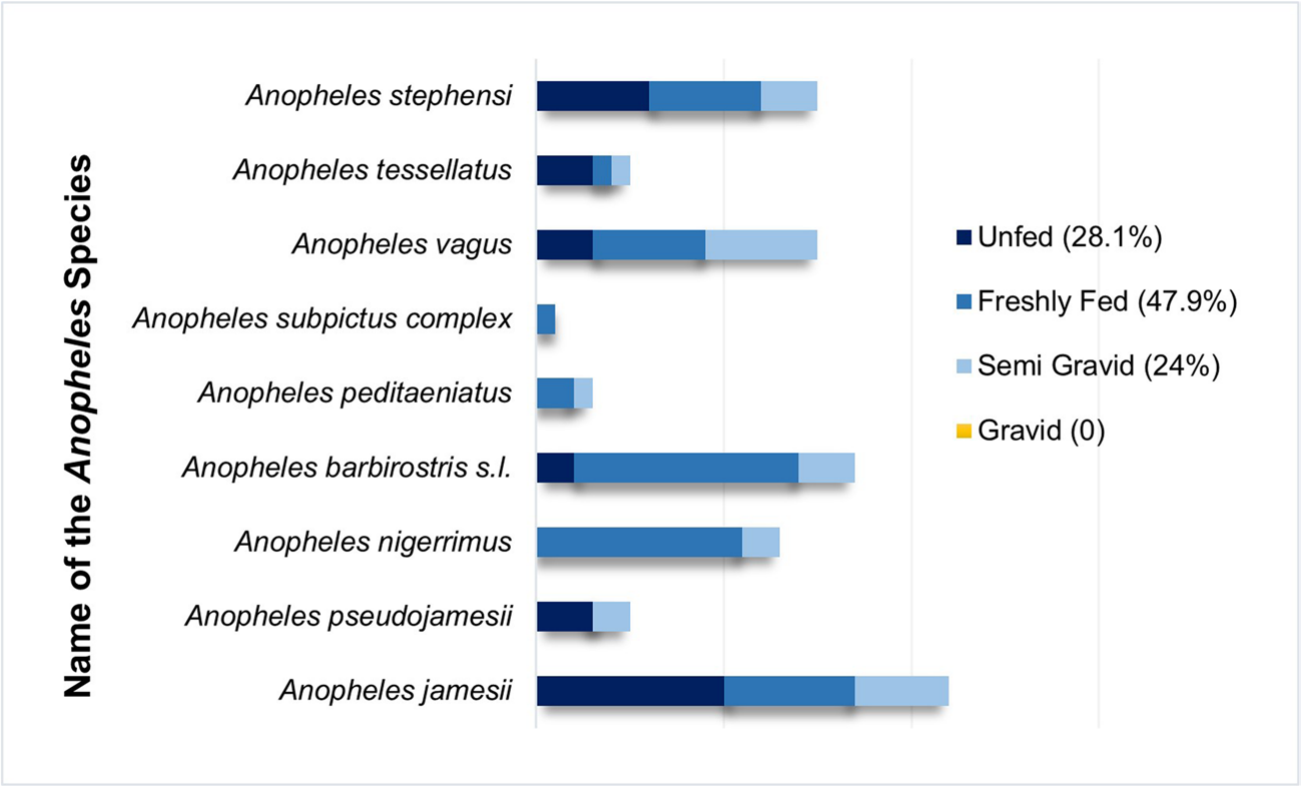
Distribution of abdominal stages across different *Anopheles* species

The abdominal conditions of overall anophelines collected comprised freshly fed (*n* = 46, 47.9%), unfed (*n* = 27, 28.1%), and semi-gravid (*n* = 23, 24%) specimens (Fig. 3). In Udupi, the majority were freshly fed (*n* = 41, 47.7%), followed by unfed (*n* = 25, 29.1%) and semi-gravid (*n* = 20, 23.3%). However, in Mangalore, freshly fed was slightly more (*n* = 5, 50%), compared to semi-gravid (*n* = 3, 30%), and unfed (*n* = 2, 20%). Moreover, a comparative analysis between the resting habitat and abdominal stages of mosquitoes revealed that cattle sheds had the highest number of mosquitoes with majority being freshly fed (50%), followed by unfed (26.2%) and semi-gravid (23.8%) (*χ*^2^ − 24.6, *d* = 1, *P* < 0.0001) (Table 4). Similarly, the comparison of abdominal condition of *An. stephensi* between the two study sites shown an equal share of freshly fed and unfed (*n* = 6 each), followed by semi-gravid (*n* = 3). PCR results showed that none of the female anopheline tested (*n* = 96) were positive for plasmodium-specific DNA.

**Table 4:** Abdominal stages of *Anopheles* mosquitoes in 3 different resting habitats

Resting habitat	Abdominal stages	*N* (%)	*χ*^2^	*P* value
Human dwellings	Unfed	2 (50)	4.26	0.0389*
	Freshly fed	2 (50)		
	Semi-gravid	0 (0)		
	Gravid	0 (0)		
Construction sites	Unfed	3 (37.5)	2.13	0.1441
	Freshly fed	2 (25)		
	Semi-gravid	3 (37.5)		
	Gravid	0 (0)		
Cattle sheds	Unfed	22 (26.2)	24.6	< 0.0001*
	Freshly fed	42 (50)		
	Semi-gravid	20 (23.8)		
	Gravid			

*N* Number of mosquitoes; %percentage of mosquitoes; *significant (*p* < 0.05)

## Discussion

Entomological surveillance is essential for implementing an evidence-based malaria control program that achieves optimal results in malaria elimination, as it is essential for comprehending mosquito bionomics, identifying spatio-temporal trends of vectors, and monitoring potential vectors and their roles in disease transmission (Killeen et al. 2018). Consequently, entomological surveillance permits malaria control programs to evaluate their performance, identify gaps and challenges, and modify strategies accordingly.

The present study was undertaken in two malaria-endemic cities of Karnataka, where no previous studies has investigated the diversity and abundance of anophelines in relation to malaria transmission. This study identified 21 distinct species of mosquitoes, *Anopheles* accounted for 5.4% in 7 sampled points, while *Culex* predominated in all collected sites. This finding is consistent with studies that have reported *Culex *as the predominant species in terms of abundance (Adugna et al. 2020; Balthazar et al. 2021; Ishwara Prasad et al. 2021).

Notably, *Cx. quinquefasciatus*, *Cx. gelidus*, and *Ae. albopictus* were the most prevalent among the culicine species. Among anophelines, *An. jamesii*, *An. barbirostris* s.l., and *An. stephensi* were the predominant species, while other species such as *An. nigerrimus* and *An. vagus* were also present in considerable number. The overall mosquito density, especially anophelines, exhibited considerable variation between seasons. This observation underscores the importance of considering seasonal fluctuations in mosquito populations when assessing their impact on malaria transmission.

*An. stephensi* that has been extensively studied in India for its crucial role in urban malaria transmission (Korgaonkar et al. 2012; Subbarao et al. 2019; Sumodan et al. 2004; Thomas et al. 2017; Tikar et al. 2011), was observed in our study to be prevalent in both districts with similar densities. Of the seven locations sampled, *An. stephensi* was more abundant in Kunjibettu, Laxmindra Nagar, and Bolar than in the other area. Similarly, the prevalence of malaria vector *An. subpictus* complex, which has been suggested to play a role in malaria transmission in Southeast Asia (Kumar et al. 2016; Kumari et al. 2013; Surendran et al. 2012), was extremely low in the present study. Additionally, the current investigations concentrated on urban settings, which resulted in a lower number of *Anopheles* species, particularly in Mangalore, compared to the study conducted by Prasad and Sreepada (2013). Although the present study revealed temporal variations in *Anopheles* species across different seasons, no significant differences in mosquito densities were observed between the two districts.

Various resting behaviours have been observed in *Anopheles* species (Subbarao et al. 2019). This study indicated a relatively lower number of *Anopheles* mosquitoes resting inside the human dwellings compared to cattle sheds. However, the density of *An. stephensi* was slightly high at construction sites, consistent with the findings of another study conducted elsewhere (Sumodan et al. 2004). This might be attributed to the species preference to breed in such habitats (Anvikar et al. 2016; Ghosh et al. 2008; Ishwara Prasad et al. 2021). Correspondingly, the high proportion of other anophelines, with the majority being freshly fed in the cattle sheds, indicated high host availability and habitat preference. Moreover, the statistical analysis demonstrated a noteworthy difference in anopheline density based on various resting preferences.

Ghosh et al. (2008) reported one out of 46 *An. stephensi* samples positive for *Plasmodium* infection, which were collected from construction sites in Mangalore, but no infection was detected in any of the anophelines collected in this study.

While these findings are preliminary, this study still contributes to our current understanding of the seasonal abundance and resting habitats of the prevalent anopheline mosquitoes in two coastal cities. Further research is warranted given the study’s limitations: (i) the low prevalence of the vector, prevented drawing a conclusion from the available samples. (ii) Although we observed the prevalence of two anopheline vectors in the study areas, a larger sample pool may be necessary to accurately estimate the actual infection rate. (iii) Additionally, the blood meal preference of anophelines could not be determined.

## Conclusion

In coastal Karnataka, the study area displays a low but concentrated transmission of malaria. To effectively eliminate malaria, it is crucial to possess a detailed knowledge about local vector species and the dynamics influencing their behaviour. Despite the current study uncovering the presence of two significant malaria vectors, long-term monitoring of these species is essential to ascertain their role in transmission. Therefore, our study underscores the necessity for ongoing research in these regions, aiming to enhance our understanding of anophelines concerning larval habitats, feeding preference, species specific characteristics, and the impact of climate factors.

## Supplementary Information

Below is the link to the electronic supplementary material.

Supplementary file1 (DOCX 15.1 KB)

Supplementary file2 (DOCX 37.6 KB)

## Data Availability

Data is provided within the manuscript and supplementary information files.

## Declarations

### Disclaimer

The funders had no role in study design, data collection and analysis, decision to publish, or preparation of the manuscript.
