# Supplementary material for: Mosquito prevalence, resting habitat preference, and Plasmodium infection status of anophelines in coastal Karnataka during the declining phase of malaria—an exploratory study
Source: Parasitol Res. 2024 Aug 22;123(8):308. doi: 10.1007/s00436-024-08322-x (PMC11341726; doi:10.1007/s00436-024-08322-x)
Supplement: Supplementary file 1 — Supplementary file1 (DOCX 15.1 KB) [file 436_2024_8322_MOESM1_ESM.docx]

**Title: Mosquito prevalence, resting habitat preference and *Plasmodium* infection status of anophelines in coastal Karnataka during the declining phase of malaria-an exploratory study**

**S1 file: PCR primer sequence for specific targeted gene**

| **Target species for 18S rRNA gene** | **Primer sequence (5’ – 3’)** |
| --- | --- |
| *Plasmodium* | F - TTAAAATTGTTGCAGTTAAAACG  R - CCTGTTGTTGCCTTAAACTTC |
| *Plasmodium falciparum* | F - TTAAACTGGTTTGGGAAAACCAAATATATT  R - ACACAATGAACTCAATCATGACTACCCGTC |
| *Plasmodium vivax* | F - CGCTTCTAGCTTAATCCACATAACTGATAC  R - ACTTCCAAGCCGAAGCAAAGAAAGTCCTTA |
