# Supplementary material for: Mosquito prevalence, resting habitat preference, and Plasmodium infection status of anophelines in coastal Karnataka during the declining phase of malaria—an exploratory study
Source: Parasitol Res. 2024 Aug 22;123(8):308. doi: 10.1007/s00436-024-08322-x (PMC11341726; doi:10.1007/s00436-024-08322-x)
Supplement: Supplementary file 2 — Supplementary file2 (DOCX 37.6 KB) [file 436_2024_8322_MOESM2_ESM.docx]

**Mosquito prevalence, resting habitat preference, and *Plasmodium* infection status of anophelines in coastal Karnataka during the declining phase of malaria-an exploratory study.**

***Corresponding author details**

**Kavitha Saravu**

Professor and Head, Department of Infectious Diseases, Kasturba Medical College, Manipal, Manipal Academy of Higher Education, Manipal, Karnataka, India-57610

**E-mail:** [kavitha.saravu@manipal.edu](mailto:kavitha.saravu@manipal.edu)

**S2 file: Variation in mean density of Anopheline collected according to habitat type**


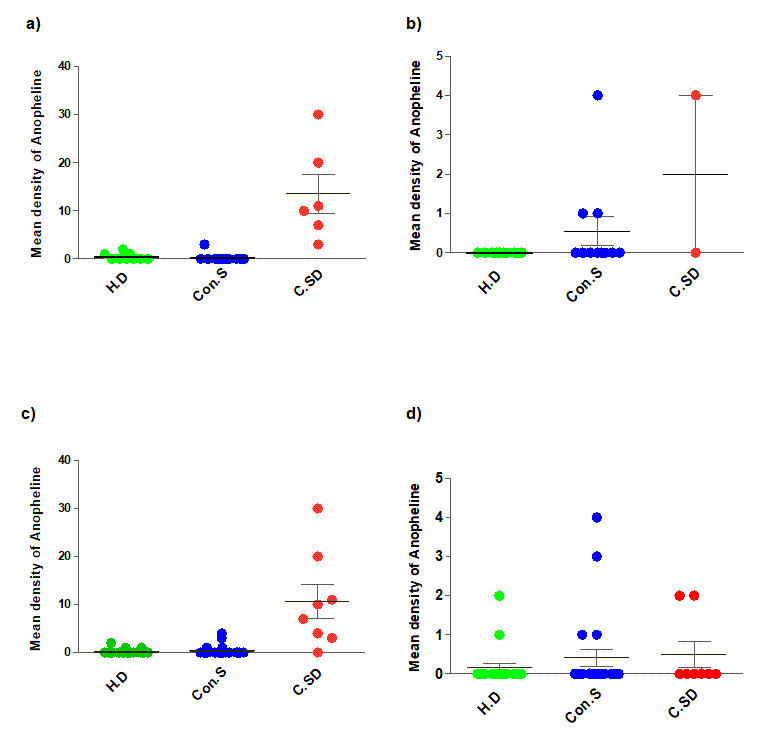
a) Udupi, b) Mangalore, c) Overall, d) overall *Anopheles stephensi*

H. D - human dwelling, Con.S - construction site, C.SD - cattle shed.
